# 

**Published:** 1854-12

**Authors:** 


We have received Harris' Dictionary of Medicine and Dental Surgery, and a neat little duodecimo, called "Nature in Disease, and other writings," by Dr. Bigelow, of Boston; both of which we shall notice in our next.

*To Correspondents.*—The communication from our friend Dr. B., of Avon, has never reached us, and as so long a time has elapsed since it was mailed, it must henceforth be ranked amon the lost manuscripts. It is a matter of no little regret that an article on which so much labor had been expended should thus fall by the wayside.

To others of our friends we would say that a certain bag wherein we deposit their favors has, by long continued depletion, become collapsed and empty. Of late it has held out like the widow's cruse of oil, but it is now again ready for the reception of visitors. We had always rather fill our journal with the fresh thoughts of American writers, than to resort to other or foreign journals for matter to fill our pages. H.

